# Effects of Parental Education Level and Economic Status on the Needs of Families of Hearing-Impaired Children in the Aural Rehabilitation Program

**Published:** 2013

**Authors:** Nazanin Eyalati, Zahra Jafari, Hassan Ashayeri, Masoud Salehi, Mohammad Kamali

**Affiliations:** 1 *Department of Rehabilitation Management, School of Rehabilitation,Tehran University of Medical Sciences,Tehran, Iran.*; 2 *Department of Basic Sciences in Rehabilitation, School of Rehabilitation, Rehabilitation Research Center, Tehran University of Medical Sciences, Tehran, Iran.*; 3 *Department of Biostatistics, School of Management and Medical Information, Tehran University of Medical Sciences,Tehran Iran.*

**Keywords:** Auditory-verbal therapy, Children, Economic status, Educational level, Parents, Needs

## Abstract

**Introduction:**

The family of each hearing-impaired child has its own cultural, social, educational, and financial background, and its own special needs. The objectives of our study were to explore the information and support needs of parents of children with severe-to-profound hearing impairment and to investigate the effects of the parents’ level of education and economic status on the score attained in the parents’-needs questionnaire.

**Materials and Methods:**

Fifty-one parents of children with severe-to-profound hearing loss (53% girls, 47% boys; mean age 47.96 months) who used the Auditory-Verbal Therapy approach were asked to complete the parents’-needs questionnaire. The questionnaire included demographic information and hearing-loss history and covered six domains which evaluated the information or support needs of parents. Parental needs with regard to different domains were evaluated separately in all participants.

**Results:**

Statistical analysis indicated that there was a significant decrease in the score attained in the parents’-needs questionnaire with increasing level of education of the child’s parents (*P*<0.0001). Furthermore, the questionnaire score decreased as the parents’ economic status increased (*P*<0.0001).

**Conclusion:**

The results of our study indicate that most parents of hearing-impaired children need adequate and appropriate information in all domains, and these findings support the positive effect of creating an appropriate educational environment by considering individualized needs. Furthermore, parents’ levels of education and economic status have a significant effect on their parents’ needs.

## Introduction

Auditory-Verbal Therapy (AVT) is an early interventional approach for children who are deaf or hard of hearing, and their families. AVT focuses on education, guidance, advocacy, family support, and the rigorous application of techniques, strategies, conditions, and procedures that promote optimal acquisition of spoken language through listening. The Auditory-Verbal therapist, in partnership with the child’s parents, forms a team with other professionals to address the child’s needs (1).

The heightened interest in the identification and noticeable development of speech and language has at times overshadowed the partnership of parents and family in the rehabilitation of the deaf and hard-of-hearing child. The level of parental involvement, as well as the quality, quantity, and timing of care services provided is essential to the psychosocial and academic development of the child and to his or her ultimate quality of life (2,3).The parents of a child without disabilities have a responsibility to meet the needs of their child and prepare an appropriate environment for his or her healthy growth and development. In additional to these responsibilities, however, the parents of a disabled child are also burdened with additional response- bilities, such as teaching specific skills and practicing rehabilitation programs. For this reason, the parents of disabled children require additional support (4,5).

Parents of deaf or hard-of-hearing children often require more information and professional support from an audiologist, a medical doctor, or other professionals. Some parents find information provided about hearing aids to be insufficient, while others find medical information difficult to understand. There is a need for the diagnostic and rehabilitation process to become clearer. According to the literature, parents of children with disabilities in general benefit from meeting other people in the same situation. Parents can meet others in similar circumstances through various ways, including the clinical list, aid organizations, or other individuals that come into contact with the children. Parents find this contact with others and the sharing of knowledge with other parents with the same worries for their child to be beneficial (6).

Parents need be able to meet the needs of the child with hearing loss as with any other family member, and need sufficient information to be able to make important decisions. Parents can even feel that they do not have the necessary skills to parent a child with a hearing loss. Importantly, the background of the parents can have an influence. Professionals need to be able to adjust to each set of parents when discussing their child’s hearing loss (6).

A study by Mukari and colleagues reported that lack of knowledge among parents can be related to factors such as the distance from and availability of support services, ethnicity, educational status, the availability of financial resources, and potentially individual levels of commitment from the parent (7).

Ozcebe and colleagues suggested that poor socioeconomic circumstances and a low level of knowledge in a family contribute to late identification of hearing loss and intervention (8). 

Musselman and Kircaali-Iftar found that the level of the parents’ education, the strength of the family’s commitment to the mode of the child’s education, the parents’ level of involvement in their child’s education, and the ability of the family to allocate roles in forwarding their goals for the child related to outcomes for children with hearing loss (9).

The objective of our study was to investigate the effect of parents’ level of education and economic status on their needs in the AVT program of severe-to-profound hearing-impaired children.

## Materials and Methods

*Participants: *This study sample consisted of 51 parents of children with severe-to-profound hearing loss (53% girls; average age 47.96 ± 17.5 months) who used the AVT approach in the Newsha Aural Rehabilitation Center in Tehran between 2009 and 2012.

This approach is dedicated to maximizing the success of every child who receives either a hearing aid or a cochlear implant. The Newsha Center offers counseling services for the parents of newly diagnosed children with hearing loss. These services include an explanation of communication choices, provision of local and national informational resources, and consultation to address concerns and questions regarding communication development in children with hearing aids or cochlear implants.* Procedure: *The instrument used in the current study for determining parental needs was a validated and reliable Persian-language version of The Needs of Hearing-Impaired Children’s Parents in AVT Questionnaire developed by Eyalati et al. in 2012 (Table 1) (10). This questionnaire was distributed by the researcher to the parents of children who used AVT. They were asked to complete and return the questionnaire to the researcher. They were also informed that participation was voluntary and that they could end their involvement at any time without any effect on the child’s services. This study was approved by the Ethics Committee of Tehran University of Medical Sciences.

**Table 1:** The Persian-language version of The Needs of Hearing-Impaired Children’s Parents in AVT Questionnaire as used in the present study

**Items**	
**General information**	
1	How children grow and develop
2	How to play with my child
5	How to talk with my child
4	How to handle my child’s behavior
**Information – hearing and hearing loss**	
5	How the normal ear hears and how the ear works
6	How my child hears
7	Cause of hearing loss
8	What hearing aids are and how they will help my child
9	What cochlear implant is and how it will help my child
10	About other types of hearing devices
11	How to keep the hearing aids (or cochlear implant) on
**Communication–—services and educational resources**	
12	How to teach my child to listen
13	Information about special services my child may need in the future
14	More time to talk with to my child’s teacher or therapist
15	Information about other conditions my child may have
16	Reading materials, videos, local, state, and national organizations and resources about hearing loss
**Family and social support**	
17	Talk with someone in my family, or a friend, about my concerns
18	Opportunities to meet with other parents of children who are deaf or hard of hearing
19	Opportunities to meet deaf and hard-of-hearing adults
20	Information about parent support groups
21	More time for myself and my family
22	Help my family members to accept hearing loss
23	Meet with a counselor who specializes in hearing loss issues
24	Explaining my child’s hearing problem to others
**Child care and community services**	
25	Help locating a good kindergarten for my child
26	Help locating a day-care program for my child
27	Help locating a doctor, dentist, etc.
28	Help with transportation
**Financial** **issues**	
29	Paying for hearing aids or cochlear implant
30	Paying for therapy
31	Paying for child care/respite care
32	Paying for other special equipment my child needs
	

	
	

Yes Info

Yes Discuss

Not Sure

No

All parents completed a history form that included their ages, socioeconomic profile (i.e., educational level and economic status) and their child’s hearing-loss history. The questionnaire included 32 items and covered six domains which evaluated the information or support needs of parents, including general information, information about hearing and hearing loss, communication services and educational resources, family and social support, child care and community services, and financial status. There were four options for each question in the different sections (no=1; not sure=2; yes, discuss=3; yes, info=4), from which respondents were expected to choose the most appropriate answer for each statement. If respondents had sufficient understanding of the information they had received, they were asked to mark ‘no’. However, if they were not sure either about the level of information or if they wanted further information, they were asked to select ‘not sure’. If they believed that they had some information and wanted to discuss it, they were required to select ‘yes, discuss’. If they felt totally uninformed and had further questions, they were asked to choose ‘yes, info’. Within 2 weeks of the initial issue, 100% of the questionnaires had been completed. 

*Data Analysis:* Statistical analysis was conducted using SPSS.17 statistical software at significant levels of P<0.05. Preliminary analysis with Kolmogorov-Smirnov tests showed a normal distribution of the data (P>0.147). The relationship between total score and each of the six domains of the questionnaire with the parents’ level of literacy and economic status was analyzed using the Wilcoxon signed-rank test, separately. Correlation between the age of the children and the questionnaire score was also determined using the Pearson correlation test.

## Results

A total of 51 questionnaires were completed appropriately. At the time of this study, the ages of the parent respondents ranged from 21 to 42 years for the mothers (mean, 30.7 ± 5.16 years) and from 24 to 50 years for the fathers (mean, 34.9 ± 5.81 years). 

**Fig 1:**
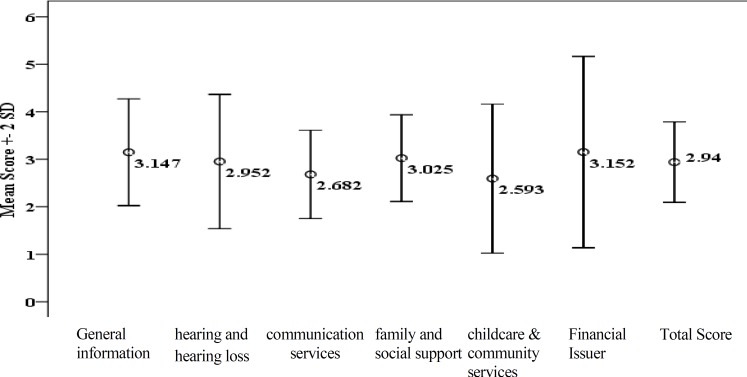
Mean (±2SD) total score and score of each of six domains of the questionnaire

Parental needs with regard to six different domains including general information, hearing and hearing loss, communication services and educational resources, family and social support, childcare and community services, and financial stats were evaluated separately in all participants (Fig 1). 

**Fig2:**
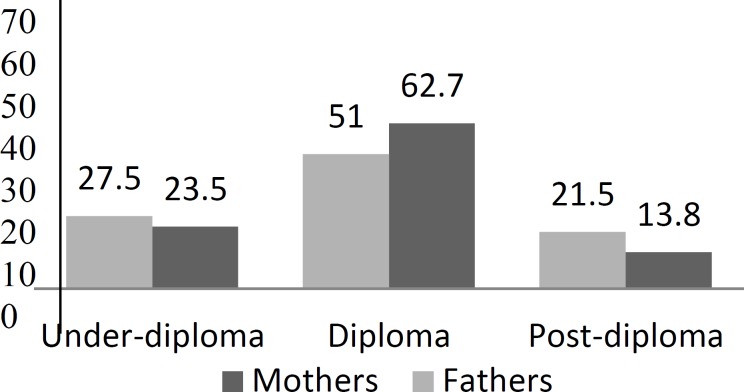
Distribution of parents’ level of education

All parents had received education to at least primary-school level (Fig 2). 

Data analysis indicated that there was a significant correlation between the total score and the score of each of the six domains of the parents’-needs-questionnaire with the parents’ level of education (Table 2).

**Table 2:** Correlation between scores of the questionnaire with the parents’ educational level and economic status.

Subtests of the Questionnaire	Educational Level		Economic Status	
	Z-Test	P-Value	Z-Test	P-Value
General information	−5.927	<0.0001	−6.140	<0.0001
Hearing and hearing loss	−6.220	<0.0001	−5.706	<0.0001
Communication services and educational resources	−5.122	<0.0001	−5.314	<0.0001
Family and social support	−6.221	<0.0001	−6.034	<0.0001
Childcare and community services	−4.029	<0.0001	−3.356	<0.0001
Financial issues	−5.018	<0.0001	−5.229	<0.0001
Questionnaire total score	−5.889	<0.0001	−6.056	<0.0001

**Fig 3:**
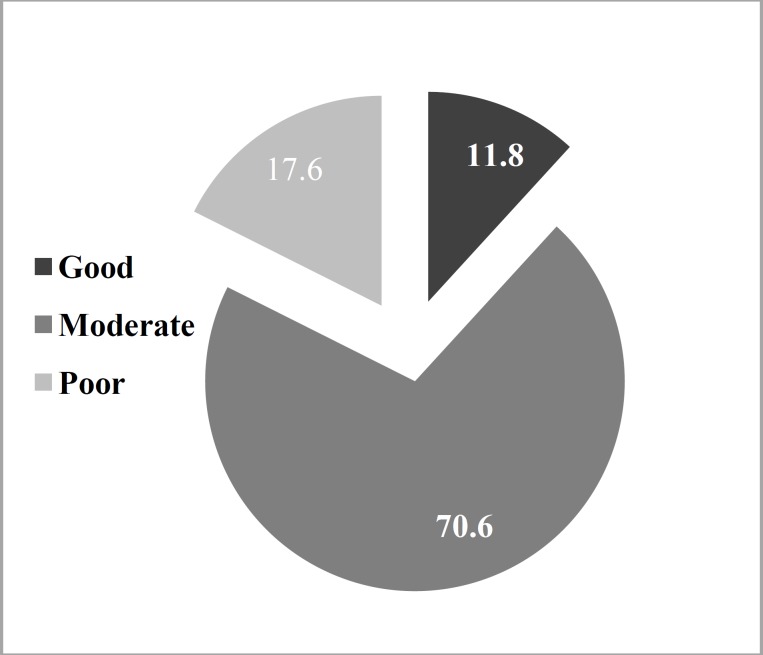
Distribution of economic status of the families

Based on parent interviews, the parent respondents were divided into three categories; poor, moderate, and of good economic status (Fig 3). 

Data analysis showed that there was a significant correlation between the total score and the score of each of the six domains of the parents’-needs questionnaire with the parents’ economic status (Table 2).

At the time of the study, the mean chronological age of the children was 47.96 months. There was no significant correlation between the age of the children and the score of the parents’-needs questionnaire (r=0.261, P=0.064).

## Discussion

Unfortunately in Iran, general knowledge about hearing loss is poor. Not only do parents of children with hearing loss need more information, but professionals who are working with children with hearing loss also need more information about hearing loss and its consequences for children’s development and behavior. In this study, analysis of parents’ answers to the questionnaire indicated that most of the parents with hearing-impaired children needed additional and appropriate information in all domains, and they suffered from inadequate knowledge to help their children and their family to accept the hearing loss. They particularly needed information in the general, family and social support, and financial domains. Possible reasons for these findings could be the lack of public awareness about the importance of child-behavior management, poor representation of disability and its effects in the media, and poor consulting systems in rehabilitation centers. The frequency and percentages of answers in the present study are close to the results of a study by Yucel and colleagues, in which the greatest parental needs were in the family and social support, child care and community services, and financial fields (11). Lutermanand Kurtzer-White reported that the greatest needs of parents when their child is diagnosed with hearing loss were opportunities to meet with other parents of children who are deaf or hard of hearing, as well as the provision of appropriate information, emotional support, and access to services (12).

The parental needs and responses to questions seem to correlate with a number of characteristics of parents, such as their background experience with hearing loss, their level of education, the age of their children, their economic status, and the presence of another hearing-impaired individual in the family. Fitzpatrick and colleagues suggested that family characteristics and their involvement in the rehabilitation process can reduce the negative effects of a late diagnosis of hearing loss (13).

In this study, there was a remarkable decrease in the score of the parents’-needs questionnaire with increasing level of parents’ education. This means that parents who had a higher level of education needed less information in any field than those with less education. Wong and colleagues reported that parents needed to be equipped with practical parenting skills and information on developmental disability. They also needed professional support to cope with caring tasks and activities; in this way, education is recommended as a fundamental strategy to enhance parents’ caring competency (14). Jeddi and colleagues also reported that parent’s levels of education and economic status have a remarkable effect on the age of cochlear implantation in hearing-impaired children (15). On the other hand, a study by Wu and colleagues found that there were no associations between parental level of education and degree of parental involvement in a program for children with hearing loss (16).

In current study, the score of the parents’-needs questionnaire decreased as the level of the parents’ economic status increased. This finding means that parents with better socioeconomic status have more information about all domains of the questionnaire. The principle reason for this could be that the parents’ socioeconomic level appears to be an important variable contributing to a child's ownership of hearing aids, the proper maintenance of the hearing aids, better access to special intervention services, and use of public welfare systems. This can be attributed to the fact that individual hearing aids are expensive, and poorer parents may not have the financial capacities to purchase them for their child. Yucel and colleagues reported that the low socioeconomic status and low level of awareness of the families, and the delays in obtaining a hearing aid device caused by economic limitations are the major factors that may contribute to the prolongation of the interval between amplification and intervention (11).

On the other hand, Mukari and colleagues found no notable differences between socioeconomic status in terms of the communication method used with the hearing-impaired children (7). Parents play a major role in the rehabilitation process. Parents of hearing-impaired children often feel there is a shortage of information about the hearing loss and its own outcomes and would often like more information with easier access. For parents it is particularly important that they are given information about the child’s future and development, although they understand that this is sometimes uncertain. Parents’ experience of treatment and services around hearing loss and hearing aids could be improved with more support and more valuable information. There is also need for more knowledge about hearing loss and hearing aids in the wider society; for example, among professionals working with children. 

Although support is important for the parents of children with hearing loss, access to support, whether it is from professionals, other parents of children with hearing loss, or family and friends may be limited (6). The constraints of this study were the number of aural rehabilitation centers and hearing-impaired children who use AVT.

## Conclusion

The results of this study indicate that most of the parents of hearing-impaired children need adequate and appropriate information in all domains tested. These findings support the positive effect of creating an appropriate educational environment by considering individualized needs. Also, exploring parental needs is very important for planning and making decisions in the rehabilitation process. 

The parents’-needs questionnaire should be used in all clinics, hospitals, and rehabilitation centers to determine the needs of parents of children with hearing dysfunction. Professionals who work on social services and healthcare systems must ensure that the best match between parental priorities and services is offered. Parental levels of education and economic status also have a noticeable effect on the rate of parents’ needs. Therefore, providing an appropriate educational environment and forming parent support groups is crucial. The public media should also present reading materials, videos, as well as information about local, state, and national organizations and resources relating to hearing loss. 
